# Prevalence of Delayed Eruption of Permanent Upper Central Incisors at a Tertiary Hospital in Riyadh, Saudi Arabia

**DOI:** 10.3390/children9111781

**Published:** 2022-11-19

**Authors:** Mannaa Aldowsari, Faisal S. Alsaif, Mohammed S. Alhussain, Bander N. AlMeshary, Naif S. Alosaimi, Saleh M. Aldhubayb, Sakher AlQahtani

**Affiliations:** 1 Department of Pediatric Dentistry and Orthodontics, College of Dentistry, King Saud University, Riyadh 12372, Saudi Arabia; 2 Private Sector, Riyadh 12613, Saudi Arabia; 3 Ministry of Health, Riyadh 12613, Saudi Arabia

**Keywords:** delayed eruption, dental age, permanent incisors, prevalence, teeth

## Abstract

Tooth eruption is defined as the axial movement of the tooth from its site of development in the alveolar bone to its functional position in the oral cavity. A delay in eruption can directly affect the accurate diagnosis, overall treatment planning, and timing of treatment. Thus, Delayed Tooth Eruption can have a significant impact on a patient’s proper health care. This study aimed to estimate the prevalence of delayed eruptions of permanent upper central incisors in Riyadh, Saudi Arabia. A retrospective study assessed panoramic radiographs of children between the ages of 6–10 years old who attended the Dental University Hospital clinics at King Saud University in Riyadh, Saudi Arabia between 2014 and 2020. The radiographs were collected and examined to detect any delayed eruption of the permanent upper central incisors. Panoramic radiographs with delayed erupted permanent upper central incisors were identified by tooth developmental stages and eruption sequence. Of the 745 radiographs, 23 (3.09%) presented at least one delayed erupted permanent upper central incisor of which boys and girls comprised 16 (69.5%) and 7 (30.4%), respectively. Retained primary teeth was the most causative factor of delayed eruption (43.4%), followed by the early loss of primary teeth (21.7%) and insufficient arch space. The present study is the first to assess the prevalence of delayed eruption of the permanent upper central incisors on a Saudi population. Boys were found to have more prevalence of delayed eruption of the permanent upper central incisors compared to girls. Clinicians should be aware of such a condition as early diagnosis of delayed eruption is essential for providing optimal dental care.

## 1. Introduction

Tooth eruption is defined as the axial movement of the tooth from its site of development in the alveolar bone to its functional position in the oral cavity [1,2,3]. The mechanism of eruption is not completely understood and still under debate [4,5]. According to Ten Cate, before the tooth reaches the functional position in the oral cavity, it passes through complex movements in three phases. Phase 1: pre-eruptive movements, where the tooth is within the alveolar bone before the time of the eruption. Phase 2: eruptive movements, where the tooth moves from an intraosseous position to its functional place in the oral cavity. Phase 3: movement, where the tooth stays in its functional place and adapts to the growth of the jaws [6]. This is different from emergence, which is the moment of appearance of any part of the tooth through the gingiva [7].

The permanent teeth usually erupt between the ages of 6 and 14 years in what is known as the mixed dentition phase. Third molars are the exception, where they start to erupt between the ages of 17 and 21 years. Knowing the eruption time of individual teeth is extremely important in treatment planning. Moreover, tooth eruption is used in the forensic field when age assessment is required [8,9].

To describe tooth eruption disorders, there are some ambiguities concerning the terminology in the literature. Although many terms are used to characterize Delayed Tooth Eruption (DTE), they all refer to two fundamental factors that influence this phenomenon: (1) expected tooth eruption time (chronologic age), as resulting from population studies, and (2) biological eruption, which is assessed by the progression of root formation. However, the chronologic age method is commonly used to identify DTE as it is easy to be used [10,11,12]. One of the most accurate chronological age estimation methods used for criminal, forensic, and anthropological purposes is dental age assessment [13]. Dental radiographs can be used as a dental age assessment tool to assess the stage of dental growth of teeth, from initial tooth mineralization and crown formation to the maturation of the root apex.

Several studies have been conducted to assess the frequency of DTE in permanent teeth. A study by Johnsen found that the lower second premolars were the most frequent permanent teeth associated with delayed eruption, followed by upper canines and upper central incisors [14]. Another study concerned with the occurrence of unerupted permanent incisors reported that the upper central incisors were the most commonly involved with delayed eruption (1.4%), followed by upper lateral incisors (0.4%), lower central incisors (0.06%), and lower lateral incisors (0.08%) [13].

Delayed eruption of maxillary permanent central incisors can be diagnosed in two ways, clinically and radiographically [13,15]. The tooth is suspected to be delayed clinically when the contralateral tooth has erupted more than six months ahead or when the sequence of eruption is disturbed [16]. A delayed eruption can also be diagnosed radiographically, by using preapical, occlusal, or panoramic radiographs, which also can be helpful in locating the position of the tooth in question along with any other developmental anomalies or pathologies (e.g., cyst and tumor) [7,10,17].

There are many local factors that have been related to the delayed eruption of the upper central incisors, such as malformation, congenitally missing, and supernumerary teeth [14,18]. Moreover, early extraction or loss of primary teeth, retained primary teeth, dilacerations, trauma, and loss of space are frequent causes as well [14,15]. Among them, dilacerations and the presence of mesiodens were the most common causes for the delayed eruption of maxillary permanent central incisors [15,19].

Delayed tooth eruption is the most encountered deviation from normal eruption time. However, in most cases, it is recognized by chance in a routine dental examination. Therefore, it is crucial that dentists be aware of this condition since early detection and intervention can aid in preventing many complications. The treatment of teeth with delayed eruption requires multidisciplinary cooperation between pediatric dentists, orthodontists, oral surgeons, and prosthodontists.

Only two studies have assessed the prevalence of delayed erupting permanent maxillary central incisors, which were 0.9% and 1.8% [13,14]. One population that has not been studied in this regard is the Saudi population. Therefore, the aim of this study is to measure the prevalence of delayed eruption of permanent maxillary central incisors in Saudi Arabia.

## 2. Materials and Methods

### 2.1. Study Sample

This was a retrospective cross-sectional study using dental panoramic radiographs of patients who attended the Dental University Hospital clinics in the medical city of King Saud University in Riyadh, Saudi Arabia, between 2014 and 2020. Ethical approval for the study has been granted by the Institutional Review Board of King Saud University (E-20-4947). A simple randomized sampling protocol was used to select the required sample size. At α = 0.05, with an effect size of 0.13 and power of 0.90, the total sample size was calculated to be at least 710 samples [13].

The inclusion criteria were children between the ages of 6 and 10 years who had good-quality digital panoramic radiographs in their hospital files. The exclusion criteria were children who did not have radiographs in their hospital records, low-quality radiographs, or who did not match the age range.

The digital panoramic radiographs were collected and examined to detect delayed eruption of the permanent maxillary central incisors. The radiographical assessment was based on the Atlas of Tooth Development and Eruption in regard to the alveolar tooth eruption timing and sequence to aid in the decision-making of the diagnosis of delayed eruption cases [20].

### 2.2. Training and Calibration

Operators attended hands-on training to identify tooth developmental stages by an expert in dental age estimation. Inter- and intra-examiner reliability was evaluated using a set of panoramic radiographs different from the main sample and by reassessment after an interval of two weeks using kappa [21].

### 2.3. Image Assessment

Radiographs were collected in an assigned radiographic reporting room in a dimmed light environment, using a graphic monitor with “PlanmecaRomexis” dental radiographic software by two observers. Simple image manipulation was used for more accurate image interpretation, such as contrast, brightness, and sharpness adjustments. A customized data collection sheet was used for data collection. The following relevant information of each patient included in this study was recorded: gender, date of birth, date of panoramic radiograph, tooth number, evidence of supernumerary teeth, medical condition, previous orthodontic treatment, and history of trauma if present. Teeth with evidence of delayed eruption were recorded based on the inclusion/exclusion criteria as follows based on stages explained in the Atlas of Tooth Development [20]:

Inclusion (regarding developmental stages):

1. Stage Rc (root complete);
2. Covered by bone (stage 1 in eruption);
3. Evidence of interruption of the eruption sequence.

Exclusion:

1. Stage A1/2 or Ac and covered by bone (stage 1 in eruption);
2. Eruption stages 2 or more.

The prevalence of delayed erupted maxillary central incisors was calculated as the number of affected patients divided by the total number of the sample.

### 2.4. Statistical Analysis

All statistical tests were performed using Statistical Package for the Social Science (SPSS) (version 23.0; IBM, Armonk, NY, USA). Statistical analysis included descriptive statistics, frequencies, and crosstabs with chi-square analysis. The confidence level was set to 95% and *p* values less than 0.05 were considered significant.

## 3. Results

The study sample based on Cochran’s Formula and data (published by Johnsen, 1977; Tan et al., 2018) was *n* = 745 radiographs, of which 364 (48.8%) were males and 374 (50.2%) were females [13,14]. The intra-examiner reliability test yielded a Cohen’s kappa of 0.9 and 0.95 for the inter-examiner reliability, which both indicate excellent reliability. The total number of cases identified with the presence of delayed eruption findings was 23 (3.09%) cases, of whom 16 (4.4%) were males and 7 (1.8%) were females (Table 1). Of all cases with delayed eruption, 18 (78%) were unilateral, and 5 (22%) presented a bilateral delayed eruption within the upper centrals. The most commonly affected tooth was the upper left central incisor with a total number of 13 cases, followed by the upper right central incisor with a total number of 12 cases either unilaterally or bilaterally (Table 2).

**Figure 1:**
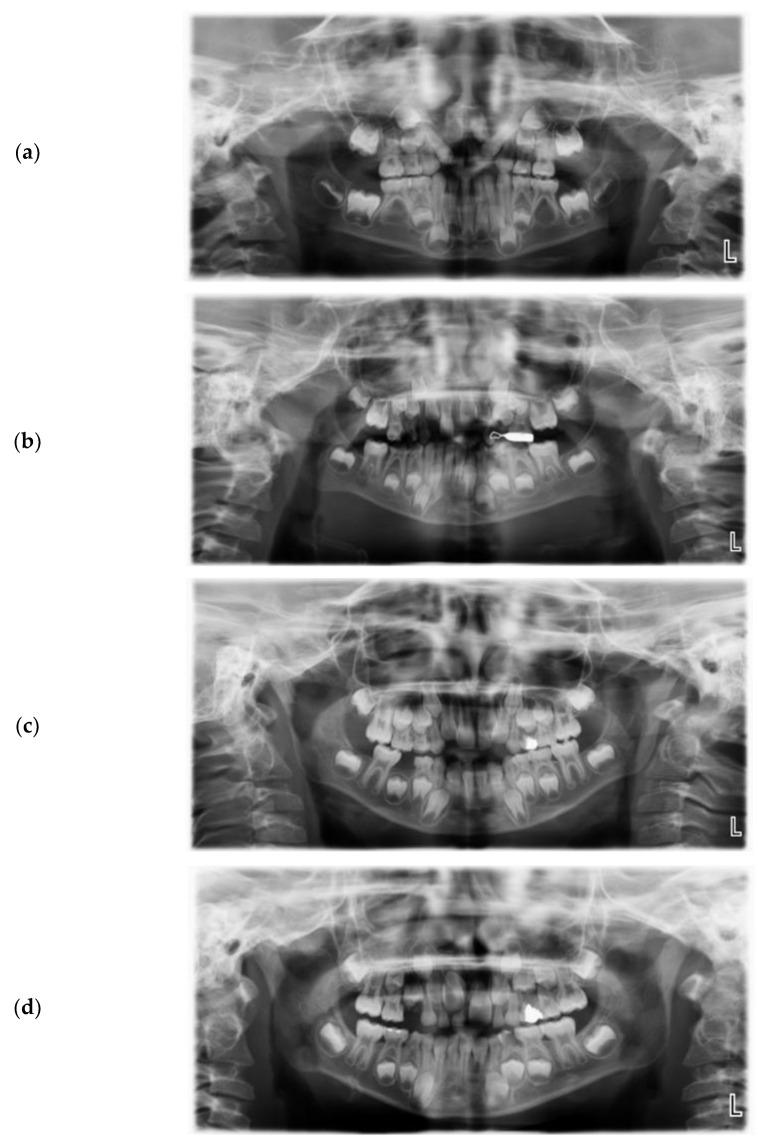
(**a**) Panoramic radiograph showing delayed eruption of permanent upper central incisor due to cleft lip and palate and insufficient space. (**b**) Delayed eruption of permanent upper central incisors due to early loss of primary upper central incisors. (**c**) Delayed eruption of permanent upper central incisors due to retained primary upper left central incisors. (**d**) Delayed eruption of permanent upper central incisors due to retained primary upper incisors and the presence of supernumerary teeth.

A retained primary tooth was found to be the most common cause of delayed eruption (10 cases (43.4%)), followed by early loss of a primary tooth (5 cases (21.7%)). (Figure 1) (Table 3).

## 4. Discussion

It is apparent that delayed eruption of maxillary central incisors can be seen in children and adolescents frequently in the dental clinic. A failure of eruption will affect the developing occlusion and potentially influence the psychological development of the child [15].

The present study aimed at measuring the prevalence of delayed eruption of permanent maxillary central incisors at a tertiary hospital in Saudi Arabia. The sample size was calculated based on previously published studies [13]. Application of this data to other populations can aid in a general expectation of relevance towards daily practice, early diagnosis, and proper management.

The radiographic assessment used in the present study was a panoramic radiograph based on the Atlas of Tooth Development and Eruption in regard to the alveolar tooth eruption timing and sequence to aid in the decision-making of the diagnosis of delayed eruption cases [20]. Similarly, the same type of radiograph was used in a study carried out on 1032 patients of the Mott Children’s Health Center in Flint, Michigan, to detect the prevalence of delayed emergence of permanent teeth as a result of local factors [14]. However, another study used combined radiographs, including panoramic, upper anterior occlusal, and periapical radiographs, for the assessment and diagnosis of delayed erupted permanent incisors of patients attending the Pediatric Dentistry and Orthodontics clinics at the Prince Philip Dental Hospital, The University of Hong Kong, Hong Kong [13].

Few studies have investigated the prevalence of delayed eruption of permanent incisors. The prevalence rate has been reported in the published studies to be between 0.9 and 1.8% [13,14]. The prevalence of delayed eruption of permanent upper central incisors in this present study was 3.09%, which shows a higher prevalence of delayed erupted permanent maxillary central incisors compared to the prevalence reported in other studies [13,14].

The number of cases of delayed eruption of permanent upper central incisors in this present study was slightly higher in boys than girls. This is in accordance with other retrospective studies, which also reported a higher prevalence of delayed erupted maxillary central incisors in boys compared to girls [13,14]. The contributing factors for delayed erupted maxillary central incisors among male patients could be explained by a greater prevalence of supernumerary teeth in boys [22,23]. Another possible reason could be the higher prevalence of malocclusion in boys compared to girls [24]. In addition, traumatic oral injuries are considered a local factor associated with delayed tooth eruption and this was found to be more common in boys compared to girls [25].

Many local factors have been associated with delayed eruption of the upper central incisors, such as malformed, congenitally missing, and supernumerary teeth. Retained and early loss of primary teeth and insufficient arch space were the highest causative factors of delayed erupted maxillary central incisors found in this present study. Contrary to our findings, Tan et al. reported that dilacerations, supernumerary teeth, and ectopic position of tooth buds are the most common causes of delayed eruption [13]. Another study reported that mesiodens, retained primary teeth, and malformed teeth as a result of trauma are the most common etiological factors associated with delayed eruption [14].

Early loss of primary teeth was highly associated with a history of traumatic events and subsequently resulted in insufficient arch space in our patients. Cleft lip and palate and the presence of supernumerary teeth were also reported in our patients as the causative factors associated with delayed erupted maxillary central incisors. A study conducted by Abdulreza et al. reported that children with cleft lips and palates showed a delay of about 0.7 years in permanent teeth development compared to non-cleft patients [26]. In addition, supernumerary teeth can delay or prevent the eruption of central incisors in 26–50% of cases [27].

The present study was limited by its retrospective design which may sometimes overlook relevant historical information. Calculating a sample size that provided representative data and randomly selecting patients to participate in the study were conducted to obtain the estimated prevalence of delayed erupting permanent maxillary central incisors in the Saudi population of Riyadh city. Although our sample randomization and size are considered statistically representative, the results might not represent the actual prevalence of the general population because the data were collected from a single dental hospital in the region.

Our findings suggest that future studies should include multiple hospitals in Riyadh city and other cities to accurately estimate the prevalence rates of such anomalies in the Saudi population. Another suggestion for future projects is to implement a prospective design to assess the prevalence of such anomalies which will help to obtain more information about the case history and allow for detailed clinical examination.

Good history taking and the correct identification of tooth developmental stages are of paramount importance in diagnosing the condition, since it can be misdiagnosed by dentists because of low-quality radiographs or the lack of awareness of the developmental variables. Dentists should be aware of this condition since early detection and intervention can help in preventing many unwanted complications.

## 5. Conclusions

Within the limitations of this retrospective cross-sectional study, it was concluded that delayed eruption was greater in boys than in girls but without achieving a statistically significant difference. Retained and early loss of primary teeth was found to be the most common cause of delayed eruption of central permanent incisors. Clinicians should be aware of such a condition as early diagnosis of delayed eruption is essential for providing optimum dental care

## Figures and Tables

**Table 1:** Frequency distribution of delayed eruption cases according to gender.

Gender	Delayed Eruption 23 (3.09%)	Normal 722 (97%)	Total *n* = 745 (100%)	*p* Value
Boys	16 (4.4)	348 (95.6)	364 (100)	*p* < 0.05
Girls	7 (1.84)	374 (98.16)	381 (100)	

**Table 2:** Frequency distribution of delayed eruption cases according to different variables.

*n* = 745	Boys	Girls	Total (100%)
Delayed Eruption	16 (70%)	7 (30%)	23
Unilateral	13 (72%)	5 (28%)	18
Bilateral	3 (60%)	2 (40%)	5

**Table 3:** Frequency distribution of delayed eruption cases according to causes.

Cause	NO. of Cases	(100%)
Retained Deciduous Tooth	10	43.47
Early Loss of Deciduous Tooth	5	21.73
Insufficient Arch Space	4	17.39
Ectopic Eruption of Another Tooth	2	8.69
Cleft Lip and Palate	1	4.34
Supernumerary Tooth	1	4.34

## Data Availability

The database is available upon request from the corresponding author.
